# Data on a coupled ENN / t-SNE model for soil liquefaction evaluation

**DOI:** 10.1016/j.dib.2020.105125

**Published:** 2020-01-16

**Authors:** Pierre Guy Atangana Njock, Shui-Long Shen, Annan Zhou, Hai-Min Lyu

**Affiliations:** a Department of Civil Engineering, School of Naval Architecture, Ocean, and Civil Engineering, Shanghai Jiao Tong University, 800 Dong Chuan Road, Minhang District, Shanghai, 200240, China; b Key Laboratory of Intelligent Manufacturing Technology (Shantou University), Ministry of Education, Department of Civil and Environmental Engineering, College of Engineering, Shantou University, Shantou, Guangdong, 515063, China; c Discipline of Civil and Infrastructure, School of Engineering, Royal Melbourne Institute of Technology (RMIT), Victoria, 3001, Australia

**Keywords:** Liquefaction, Database, CPT, Neural network

## Abstract

The data presented in this paper pertain to case records of liquefaction potential surveys in earthquake prone areas. Field performances of 219 sites obtained from various regions (U.S.A, Japan, Turkey, China, Canada, etc …) are put on display. Specifically, this database consists of 253 cone penetration test (CPT) field records, among which 72 cases that did not liquefied and 181 cases that liquefied. In total, 10 principal variables are tabulated including the earthquake magnitude, maximum ground surface acceleration, depth, water depth, total overburden stress, effective overburden stress, Cone Penetration Test (CPT) tip resistance, CPT friction ratio, fines content, shear stress ratio. These data were arbitrarily split into a testing set of 53 cases and a training set of 200 cases. These field observations are compared to prediction values of liquefaction potential assessed using the evolutionary neural network proposed for “Evaluation of soil liquefaction with AI technology incorporating a coupled ENN/t-SNE model” [1].

**Table:** Specifications Table

Subject area	Geotechnical Engineering and Engineering Geology
Specific subject area	Geotechnical engineering is the science that explains mechanics of soil and rock and investigate the subsurface and geologic conditions
Type of data	Table
How data was acquired	Survey, Cone penetration test, artificial intelligence simulation
Data format	Raw and analyzed
Parameters for data collection	The specimens used to obtain the different soil characteristics provide in this paper were gathered from both, sites that have experienced soil liquefaction and sites that have never experienced soil liquefaction.
Description of data collection	Geotechnical surveys (Cone Penetration Test) and laboratory works such as grain size analysis (to obtain the fines content) were necessary to determine the value of the different variables.
Data source location	Japan (Niigata, Hyogo, Tohoku), New Zealand (Matata, South Island, Christchurch), USA (California, San Francisco, Mexico, San Fernando), China (Tangshan), Turkey (Duzce), Canada (Victoria)
Data accessibility	The relevant raw data can be found in this article (See Table 1 in supplementary material & Table 2) and in the supplement excel file (*database.xlsx)*.
Related research article	This article is submitted as companion paper of: Atangana Njock, P. G., Shen, S. L., Zhou, A. N., Lyu, H. M., Evaluation of soil liquefaction with AI technology incorporating a coupled ENN/t-SNE model. Soil Dynamics and Earthquake Engineering 2020, 130, 105988. https://doi.org/10.1016/j.soildyn.2019.105988*.*

**Table:** 

**Value of the Data** • It takes account of adding data from recent earthquakes • It includes revised interpretations made regarding the earthquake loading or site characteristics • The database is large enough for artificial intelligence-based analysis of liquefaction phenomenon.

## 1 Data description

The database in this article (See Table 1 in supplementary material & Table 2) was initially compiled by Boulanger and Idriss [2]. The liquefaction field data present in this paper are divided into training set (Table 1 in supplementary material) and testing set (Table 2). The rows correspond to the 253 different earthquake sites where the data were collected. Besides, 10 variables describing the earthquake and site characteristics that may have affected soil liquefaction triggering were tabulated in different columns. These variables are the earthquake magnitude, maximum ground surface acceleration, depth, water depth, total overburden stress, effective overburden stress, Cone Penetration Test (CPT) tip resistance, CPT friction ratio, fines content, shear stress ratio. Apart from these variables, the field observations (which possess181 cases that liquefied and 72 cases that did not liquefied) as well as the prediction values of the ENN are also presented. For this two columns in particular, “Yes = 1” denotes the liquefaction occurrence, while “No = 0” means “No” liquefaction was observed.

**Table 2:** Testing set.

No	Earthquake	Site	M_v_	a_max_ (g)	d (m)	dw (m)	σvc (kPa)	σ′vc (kPa)	qc (MPa)	Rf (%)	FC (%)	(τav/σ′)	Field (Yes = 1, No = 0)	ENN (Yes = 1, No = 0)
201	1964 M = 7.6 Niigata - June 16	F - Showa Bridge (right bank)	7.6	0.162	5.5	1.7	98	61	12.4	1.3	5	0.163	0	0
202	1976 M = 7.6 Tangshan - July 27	T10 - Tangshan	7.6	0.64	5.3	1.5	95	57	6	1.69	4	0.664	1	1
203	1979 M = 6.5 Imperial Valley - Oct 15	Radio Tower - Unit B (R2)	6.53	0.2	3.5	2.1	62	49	1.4	0.61	64	0.159	1	0
204	1980 M = 6.3 Victoria (Mexicali) - June 9	Delta Site 3p	6.33	0.19	3	2.2	54	46	1.9	0.97	52	0.139	1	1
205	1981 M = 5.9 WestMorland - April 26	Wildlife B	5.9	0.26	4.8	1.2	90	54	5.33	1.5	30	0.258	1	1
206	1983 M = 7.7 Nihonkai-Chubu - May 26	Akita A	7.7	0.17	1.8	0.8	33	23	2.2	1.15	5	0.156	1	1
207	1983 M = 6.9 Borah Peak - Oct 28	Whiskey Springs Site 3	6.88	0.5	7.3	6.8	129	124	8.19	2.62	20	0.306	1	1
208	1987 M = 6.6 Edgecumbe, NZ - Mar 2	Awaroa Farm AWA001	6.6	0.37	2.8	1.2	51	35	6.73	1.11	35	0.341	1	1
209	1987 M = 6.6 Edgecumbe, NZ - Mar 2	Brady Farm BDY001	6.6	0.4	7.2	1.7	134	79	2.31	1	30	0.392	1	1
210	1987 M = 6.6 Edgecumbe, NZ - Mar 2	Edgecumbe Pipe Breaks	6.6	0.39	5.5	2.5	100	71	5.95	0.39	5	0.331	1	1
211	1987 M = 6.6 Edgecumbe, NZ - Mar 2	Gordon Farm GDN001	6.6	0.43	2.7	0.5	50	29	3.34	0.65	1	0.48	1	1
212	1987 M = 6.5 Superstition Hills 02 - Nov 24	Radio Tower - Unit B (R4)	6.54	0.18	2.4	2.1	42	39	6.4	1.08	18	0.123	0	0
213	1989 M = 6.9 Loma Prieta - Oct 18	State Beach Kiosk	6.93	0.28	3.6	1.8	64	47	4.75	0.49	1	0.24	1	1
214	1989 M = 6.9 Loma Prieta - Oct 18	State Beach Pathway	6.93	0.28	3.3	2.5	58	50	9	0.34	1	0.206	1	1
215	1989 M = 6.9 Loma Prieta - Oct 18	State Beach (UC-18)	6.93	0.28	3.9	3.4	68	63	16.3	0.28	1	0.188	0	0
216	1989 M = 6.9 Loma Prieta - Oct 18	Sandhold Road SI-2	6.93	0.28	2.5	1.8	44	38	7.7	0.27	2	0.211	1	1
217	1989 M = 6.9 Loma Prieta - Oct 18	Sandhold Road SI-2	6.93	0.28	8	1.8	148	87	30	0.27	4	0.276	0	0
218	1989 M = 6.9 Loma Prieta - Oct 18	Sandhold Road SI-4	6.93	0.28	1.4	1.4	24	24	3	0.36	4	0.181	1	1
219	1989 M = 6.9 Loma Prieta - Oct 18	Sandhold Road SI-4	6.93	0.28	3.5	1.4	64	43	10	0.3	4	0.26	0	0
220	1989 M = 6.9 Loma Prieta - Oct 18	Sandhold Road SI-5	6.93	0.28	5	1.8	92	60	9	0.3	4	0.261	1	1
221	1989 M = 6.9 Loma Prieta - Oct 18	Sandhold Road SI-5	6.93	0.28	7	1.8	129	78	19	0.3	1	0.274	0	0
222	1989 M = 6.9 Loma Prieta - Oct 18	Sandhold Road UC-3	6.93	0.28	2.5	1.7	45	37	8.7	0.35	4	0.216	0	0
223	1989 M = 6.9 Loma Prieta - Oct 18	Sandhold Road UC-2	6.93	0.28	2	1.7	35	32	10.4	0.32	4	0.196	0	0
224	1989 M = 6.9 Loma Prieta - Oct 18	Sandhold Road UC-6	6.93	0.28	6.5	1.7	120	73	18.2	0.17	1	0.275	0	0
225	1989 M = 6.9 Loma Prieta - Oct 18	MBARI No. 3 (RC-5)	6.93	0.28	3.5	1.8	63	47	15.5	0.15	1	0.239	0	0
226	1999 M = 7.5 Kocaeli - Aug 17	Soccer Field SF-5	7.51	0.37	1.8	1	33	25	1.28	1.2	16	0.314	1	1
227	1999 M = 7.5 Kocaeli - Aug 17	Police Station Site, PS-1	7.51	0.4	2.3	1	42	29	1.14	1.95	12	0.368	1	1
228	1999 M = 7.5 Kocaeli - Aug 17	Yalova Harbor YH-3	7.51	0.37	3.6	1	67	41	5.4	0.43	9	0.38	1	1
229	1999 M = 7.6 Chi-Chi - Sept 20	Nantou Site C-7	7.62	0.38	3.5	1	65	40	1.1	0.58	60	0.39	1	1
230	2010 M = 7.1 Darfield - Sept 4	KAS-20	7	0.211	4.3	1.6	79	53	4.59	0.33	6	0.196	1	1
231	2011 M-6.2 Christchurch - Feb 22	KAS-20	6.2	0.172	4.3	1.6	79	53	4.59	0.33	6	0.157	1	1
232	2011 M-6.2 Christchurch - Feb 22	KAS-40	6.2	0.177	2.3	1.9	41	36	3.52	0.35	8	0.125	1	1
233	2010 M = 7.1 Darfield - Sept 4	SNB-01	7	0.17	3.6	2	66	50	5.37	0.65	9	0.141	1	1
234	2011 M-6.2 Christchurch - Feb 22	SNB-01	6.2	0.351	3.6	2	66	50	5.37	0.65	9	0.287	1	1
235	2010 M = 7.1 Darfield - Sept 4	NBT-02	7	0.17	5.8	2	107	70	4.86	0.53	12	0.157	1	0
236	2011 M-6.2 Christchurch - Feb 22	NBT-02	6.2	0.354	5.8	2	107	70	4.86	0.53	12	0.318	1	1
237	2010 M = 7.1 Darfield - Sept 4	NBT-03	7	0.168	8.6	2.4	162	101	6.02	0.54	11	0.155	1	1
238	2011 M-6.2 Christchurch - Feb 22	NBT-03	6.2	0.346	8.6	2.4	162	101	6.02	0.54	11	0.304	1	1
239	2011 M-6.2 Christchurch - Feb 22	RCH-14	6.2	0.339	4.5	2.3	81	60	2.38	0.03	7	0.279	1	1
240	2010 M = 7.1 Darfield - Sept 4	Z1-3	7	0.217	6.1	1.4	116	70	5.15	1.13	26	0.218	1	1
241	2011 M-6.2 Christchurch - Feb 22	Z1-3	6.2	0.455	6.1	1.4	116	70	5.15	1.13	26	0.444	1	1
242	2010 M = 7.1 Darfield - Sept 4	Z2-11	7	0.21	2.8	1	51	34	6.42	1.09	11	0.201	0	0
243	2011 M-6.2 Christchurch - Feb 22	Z2-11	6.2	0.447	2.8	1	51	34	6.42	1.09	11	0.422	1	1
244	2011 M = 9.0 Tohoku - Mar 11	Hinode ES	9	0.199	2.8	1.2	51	36	3.4	0.7	25	0.187	1	1
245	2011 M = 9.0 Tohoku - Mar 11	Irifune NS	9	0.256	6.3	1.6	117	71	2	1.5	66	0.276	1	1
246	1989 M = 6.9 Loma Prieta - Oct 18	Radovich (RAD-99)	6.93	0.38	6	4.1	110	91	5	0.72	18	0.276	1	1
247	2011 M-6.2 Christchurch - Feb 22	KAN-19	6.2	0.187	3.7	0.8	70	42	7.75	0.53	2	0.194	0	0
248	1964 M = 7.6 Niigata - June 16	D - Kawagisho-cho	7.6	0.162	4.4	1.1	82	49	3.98	1	3	0.17	1	1
249	1976 M = 7.6 Tangshan - July 27	Y29 - Tangshan	7.6	0.12	3.3	1	61	38	0.97	0.93	75	0.122	1	1
250	1987 M = 6.6 Edgecumbe, NZ - Mar 2	Landing Rd. Bridge LRB007	6.6	0.27	5.5	1.2	102	60	5.99	0.32	1	0.279	1	1
251	1987 M = 6.2 Superstition Hills 01 - Nov 24	Wildlife B	6.22	0.133	4.8	1.2	90	54	5.33	1.5	30	0.133	0	0
252	1989 M = 6.9 Loma Prieta - Oct 18	Marine Lab (C2)	6.93	0.28	9.8	2.2	182	107	3.8	1.47	27	0.266	1	1
253	1999 M = 7.5 Kocaeli - Aug 17	Adapazari Site E	7.51	0.4	2.3	0.5	42	25	2	0.41	2	0.436	1	1

## 2 Experimental design, materials, and methods

The method used to acquire the liquefaction data provided below (Table 1 in supplementary material & Table 2) mainly consists of Cone Penetration Test (CPT). The CPT is actually one of the leading indices used to evaluate the characteristics of a soil and its susceptibility to liquefaction. Basically, a cone penetration test rig drives a steel cone into the ground until the cone reaches a hard layer [3]. The steel cone contains an electronic measuring system that records liquefaction parameters such as tip resistance and sleeve friction [4,5].

As for the approach used to obtain the ‘ENN values’, an artificial intelligence approach, specifically, an evolutionary neural network model was adopted to predict the occurrence of soil liquefaction in the earthquake prone areas (see ‘Specification Table’- Data source location). As a classification approach, this model returns the value 1 in the case of liquefaction occurrence, and the value zero otherwise. For further details, the reader can refer to the linked research article (Atangana Njock et al., 2020) [1].
