# Supplementary material for: Data on a coupled ENN / t-SNE model for soil liquefaction evaluation
Source: Data Brief. 2020 Jan 16;29:105125. doi: 10.1016/j.dib.2020.105125 (PMC7005414; doi:10.1016/j.dib.2020.105125)
Supplement: Multimedia component 3 [file mmc3.docx]

**Table 1:** Training set

| **No** | **Earthquake** | **Site** | **M_v_** | **a_max_ (g)** | **d**  **(m)** | **dw**  **(m)** | **σvc (kPa)** | **σ'vc (kPa)** | **qc (MPa)** | **Rf (%)** | **FC**  **(%)** | **(τav/σ')** | **Field (Yes=1, No=0)** | **ENN (Yes=1, No=0)** |
| --- | --- | --- | --- | --- | --- | --- | --- | --- | --- | --- | --- | --- | --- | --- |
| 1 | 1964 M=7.6 Niigata ‐ June 16 | E ‐ Showa bridge (left bank) | 7.6 | 0.162 | 3.1 | 1.4 | 56 | 40 | 3 | 1.02 | 5 | 0.147 | 1 | 1 |
| 2 | 1968 Inangahua, New Zealand ‐ May 23 | Three Channel Flat | 7.2 | 0.6 | 2.9 | 3.1 | 50 | 50 | 2 | 0.51 | 18 | 0.382 | 1 | 1 |
| 3 |  | Reedy’s Farm | 7.2 | 0.3 | 1.4 | 1.4 | 24 | 24 | 8 | 0.26 | 16 | 0.194 | 1 | 1 |
| 4 | 1975 M=7.0 Haicheng ‐ Feb 4 | Chemical Fiber Site | 7 | 0.3 | 3 | 1.5 | 54 | 40 | 1.46 | 1.37 | 60 | 0.261 | 0 | 0 |
| 5 |  | 17th Middle School | 7 | 0.3 | 3.5 | 1 | 65 | 40 | 2.35 | 1.18 | 60 | 0.304 | 0 | 0 |
| 6 | 1976 M=7.6 Tangshan ‐ July 27 | T1 ‐ Tangshan | 7.6 | 0.64 | 6.2 | 3.7 | 109 | 85 | 6.4 | 2.3 | 5 | 0.51 | 1 | 1 |
| 7 |  | T4 ‐ Tangshan | 7.6 | 0.64 | 4.7 | 1.1 | 84 | 49 | 12 | 1.09 | 4 | 0.693 | 0 | 0 |
| 8 |  | T6 ‐ Tangshan | 7.6 | 0.64 | 2.7 | 1.5 | 48 | 36 | 7.6 | 0.86 | 6 | 0.545 | 1 | 1 |
| 9 |  | T7 ‐ Tangshan | 7.6 | 0.64 | 3.4 | 3 | 59 | 55 | 4.2 | 1.57 | 41 | 0.437 | 1 | 1 |
| 10 |  | T8 ‐ Tangshan | 7.6 | 0.64 | 6.3 | 2.2 | 112 | 72 | 10 | 0.91 | 3 | 0.615 | 1 | 1 |
| 11 |  | T9 ‐ Tangshan | 7.6 | 0.64 | 4.8 | 1.1 | 86 | 50 | 8.5 | 0.95 | 10 | 0.695 | 0 | 0 |
| 12 |  | T22 ‐ Tangshan | 7.6 | 0.25 | 2.3 | 0.8 | 42 | 27 | 45 | 0.38 | 5 | 0.247 | 1 | 0 |
| 13 |  | T30 ‐ Tangshan | 7.6 | 0.2 | 6.3 | 2.5 | 115 | 78 | 14.18 | 0.38 | 2 | 0.182 | 0 | 0 |
| 14 |  | T32 ‐ Tangshan | 7.6 | 0.18 | 3.3 | 2.3 | 58 | 48 | 3.3 | 0.39 | 5 | 0.137 | 1 | 1 |
| 15 |  | T36 ‐ Tangshan | 7.6 | 0.16 | 7.4 | 2.3 | 135 | 86 | 6.6 | 0.39 | 2 | 0.154 | 0 | 0 |
| 16 |  | Y24 ‐ Tangshan | 7.6 | 0.12 | 4 | 0.2 | 75 | 38 | 1.8 | 0.75 | 75 | 0.15 | 1 | 1 |
| 17 |  | Y28 ‐ Tangshan | 7.6 | 0.12 | 2 | 0.2 | 37 | 20 | 1.38 | 0.78 | 75 | 0.146 | 1 | 1 |
| 18 | 1979 M=6.5 Imperial Valley ‐ Oct 15 | Kornbloom Rd (K4) | 6.53 | 0.13 | 3.9 | 2.7 | 69 | 57 | 2.14 | 2.12 | 85 | 0.097 | 0 | 0 |
| 19 |  | McKim Ranch ‐ Unit A (M7) | 6.53 | 0.51 | 3.2 | 1.5 | 58 | 41 | 3.22 | 0.8 | 20 | 0.448 | 1 | 1 |
| 20 |  | Radio Tower ‐ Unit B (R4) | 6.53 | 0.2 | 2.4 | 2.1 | 42 | 39 | 6.4 | 1.08 | 18 | 0.137 | 0 | 0 |
| 21 | 1980 M=6.3 Victoria (Mexicali) ‐ June 9 | Delta Site 2 | 6.33 | 0.19 | 2.7 | 2.2 | 48 | 43 | 3.1 | 0.04 | 8 | 0.133 | 1 | 1 |
| 22 | 1980 M=6.3 Victoria (Mexicali) ‐ June 9 | Delta Site 3 | 6.33 | 0.19 | 2.9 | 2 | 52 | 43 | 1.7 | 0.79 | 51 | 0.144 | 1 | 1 |
| 23 | 1980 M=6.3 Victoria (Mexicali) ‐ June 9 | Delta Site 4 | 6.33 | 0.19 | 2.3 | 2 | 41 | 38 | 3 | 0.81 | 32 | 0.13 | 1 | 1 |
| 24 | 1981 M=5.9 WestMorland ‐ April 26 | Kornbloom Rd (K4) | 5.9 | 0.32 | 3.9 | 2.7 | 69 | 57 | 2.14 | 2.12 | 85 | 0.235 | 1 | 1 |
| 25 | 1981 M=5.9 WestMorland ‐ April 26 | McKim Ranch ‐ Unit A (M7) | 5.9 | 0.09 | 3.2 | 1.5 | 58 | 41 | 3.22 | 0.8 | 20 | 0.078 | 0 | 0 |
| 26 | 1981 M=5.9 WestMorland ‐ April 26 | Radio Tower ‐ Unit B (R2) | 5.9 | 0.2 | 3.5 | 2.1 | 62 | 49 | 1.4 | 0.61 | 64 | 0.157 | 1 | 1 |
| 27 | 1981 M=5.9 WestMorland ‐ April 26 | Radio Tower ‐ Unit B (R4) | 5.9 | 0.2 | 2.4 | 2.1 | 42 | 39 | 6.4 | 1.08 | 18 | 0.136 | 0 | 0 |
| 28 | 1983 M=7.7 Nihonkai‐Chubu ‐ May 26 | Akita B | 7.7 | 0.17 | 4.5 | 1 | 84 | 49 | 2.1 | 0.74 | 5 | 0.182 | 1 | 1 |
| 29 | 1983 M=7.7 Nihonkai‐Chubu ‐ May 26 | Akita C | 7.7 | 0.17 | 2.9 | 2.4 | 52 | 47 | 3.9 | 0.99 | 5 | 0.12 | 0 | 0 |
| 30 | 1983 M=6.9 Borah Peak ‐ Oct 28 | Pence Ranch 1 & 2 | 6.88 | 0.3 | 2.8 | 1.6 | 50 | 38 | 4.91 | 1.4 | 2 | 0.249 | 1 | 1 |

**Table 1** (continued)

| **No** | **Earthquake** | **Site** | **M_v_** | **a_max_ (g)** | **d**  **(m)** | **dw**  **(m)** | **σvc (kPa)** | **σ'vc (kPa)** | **qc (MPa)** | **Rf (%)** | **FC**  **(%)** | **(τav/σ')** | **Field (Yes=1, No=0)** | **ENN (Yes=1, No=0)** |
| --- | --- | --- | --- | --- | --- | --- | --- | --- | --- | --- | --- | --- | --- | --- |
| 31 | 1983 M=6.9 Borah Peak ‐ Oct 28 | Whiskey Springs Site 1 | 6.88 | 0.5 | 2.4 | 0.8 | 44 | 29 | 5.69 | 1.85 | 20 | 0.493 | 1 | 1 |
| 32 | 1983 M=6.9 Borah Peak ‐ Oct 28 | Whiskey Springs Site 2 | 6.88 | 0.5 | 3.4 | 2.4 | 60 | 51 | 7 | 2.88 | 30 | 0.372 | 1 | 1 |
| 33 | 1987 M=6.6 Edgecumbe, NZ ‐ Mar 2 | Gordon Farm GDN002 | 6.6 | 0.37 | 1.9 | 0.9 | 35 | 25 | 9.2 | 0.33 | 1 | 0.33 | 0 | 0 |
| 34 | 1987 M=6.6 Edgecumbe, NZ ‐ Mar 2 | James Street Loop JSL007 | 6.6 | 0.28 | 5.1 | 1.2 | 93 | 54 | 6 | 0.51 | 1 | 0.291 | 1 | 1 |
| 35 | 1987 M=6.6 Edgecumbe, NZ ‐ Mar 2 | Keir Farm KER001 | 6.6 | 0.31 | 8 | 2.5 | 148 | 94 | 7.2 | 0.31 | 5 | 0.277 | 1 | 1 |
| 36 | 1987 M=6.6 Edgecumbe, NZ ‐ Mar 2 | Morris Farm MRS001 | 6.6 | 0.42 | 7.8 | 1.6 | 144 | 84 | 7.51 | 0.38 | 5 | 0.413 | 1 | 1 |
| 37 | 1987 M=6.6 Edgecumbe, NZ ‐ Mar 2 | Morris Farm MRS003 | 6.6 | 0.41 | 5.9 | 2.1 | 109 | 72 | 7.91 | 0.32 | 5 | 0.372 | 0 | 1 |
| 38 | 1987 M=6.6 Edgecumbe, NZ ‐ Mar 2 | Robinson Farm East Side | 6.6 | 0.44 | 3.8 | 0.8 | 70 | 41 | 3.6 | 0.34 | 5 | 0.471 | 1 | 1 |
| 39 | 1987 M=6.6 Edgecumbe, NZ ‐ Mar 2 | Robinson Farm West Side | 6.6 | 0.44 | 1.9 | 0.6 | 35 | 22 | 3.61 | 0.03 | 5 | 0.44 | 1 | 1 |
| 40 | 1987 M=6.6 Edgecumbe, NZ ‐ Mar 2 | Sewage Pumping Station | 6.6 | 0.26 | 5 | 1.3 | 93 | 56 | 3.98 | 0.31 | 5 | 0.26 | 1 | 1 |
| 41 | 1987 M=6.6 Edgecumbe, NZ ‐ Mar 2 | Whakatane Board Mill | 6.6 | 0.27 | 4.4 | 1.4 | 80 | 51 | 3 | 1.03 | 5 | 0.26 | 0 | 1 |
| 42 | 1987 M=6.6 Edgecumbe, NZ ‐ Mar 2 | Whakatane Hospital HSP001 | 6.6 | 0.26 | 4.7 | 4.4 | 83 | 80 | 13.62 | 0.49 | 5 | 0.165 | 0 | 0 |
| 43 | 1987 M=6.6 Edgecumbe, NZ ‐ Mar 2 | Whakatane Pony Club | 6.6 | 0.27 | 4.1 | 2.4 | 74 | 57 | 4.3 | 0.28 | 10 | 0.217 | 1 | 1 |
| 44 | 1987 M=6.2 Superstition Hills 01 ‐ Nov 24 | Radio Tower ‐ Unit B (R2) | 6.22 | 0.09 | 3.5 | 2.1 | 62 | 49 | 1.4 | 0.61 | 64 | 0.071 | 0 | 0 |
| 45 | 1987 M=6.5 Superstition Hills 02 ‐ Nov 24 | Kornbloom Rd (K4) | 6.54 | 0.174 | 3.9 | 2.7 | 69 | 57 | 2.14 | 2.12 | 85 | 0.129 | 0 | 0 |
| 46 | 1987 M=6.5 Superstition Hills 02 ‐ Nov 24 | Radio Tower ‐ Unit B (R2) | 6.54 | 0.2 | 3.5 | 2.1 | 62 | 49 | 1.4 | 0.61 | 64 | 0.159 | 0 | 0 |
| 47 | 1989 M=6.9 Loma Prieta ‐ Oct 18 | Woodward Marine (15‐A) | 6.93 | 0.28 | 2.9 | 1.3 | 53 | 37 | 5.1 | 0.22 | 3 | 0.253 | 1 | 1 |
| 48 | 1989 M=6.9 Loma Prieta ‐ Oct 18 | Woodward Marine (14‐A) | 6.93 | 0.28 | 3.6 | 1.2 | 66 | 43 | 7.8 | 0.05 | 3 | 0.273 | 1 | 1 |
| 49 | 1989 M=6.9 Loma Prieta ‐ Oct 18 | Marine Lab (UC‐1) | 6.93 | 0.28 | 11 | 2.4 | 204 | 120 | 4.6 | 1.05 | 30 | 0.26 | 1 | 1 |
| 50 | 1989 M=6.9 Loma Prieta ‐ Oct 18 | Marine Lab (UC‐7) | 6.93 | 0.28 | 8.3 | 1.4 | 155 | 87 | 4.3 | 1.87 | 30 | 0.288 | 1 | 1 |
| 51 | 1989 M=6.9 Loma Prieta ‐ Oct 18 | Marine Lab (UC‐8) | 6.93 | 0.28 | 8.6 | 1.3 | 160 | 89 | 4.3 | 1.35 | 30 | 0.29 | 1 | 1 |
| 52 | 1989 M=6.9 Loma Prieta ‐ Oct 18 | Marine Lab (C3) | 6.93 | 0.28 | 4.4 | 1.5 | 81 | 52 | 8.2 | 0.28 | 3 | 0.268 | 1 | 1 |
| 53 | 1989 M=6.9 Loma Prieta ‐ Oct 18 | Marine Lab (C4) | 6.93 | 0.28 | 5.5 | 2.8 | 99 | 73 | 1.9 | 0.21 | 3 | 0.232 | 1 | 1 |
| 54 | 1989 M=6.9 Loma Prieta ‐ Oct 18 | Model Airport (AIR‐18) | 6.93 | 0.26 | 2.8 | 2.4 | 50 | 47 | 1.8 | 1.03 | 23 | 0.177 | 1 | 1 |
| 55 | 1989 M=6.9 Loma Prieta ‐ Oct 18 | Model Airport (AIR‐21) | 6.93 | 0.26 | 2.6 | 2.4 | 47 | 45 | 2.1 | 0.41 | 5 | 0.173 | 1 | 1 |
| 56 | 1989 M=6.9 Loma Prieta ‐ Oct 18 | Miller Farm (CMF‐3) | 6.93 | 0.36 | 5.8 | 4.9 | 105 | 97 | 3.57 | 0.41 | 27 | 0.237 | 1 | 1 |
| 57 | 1989 M=6.9 Loma Prieta ‐ Oct 18 | Miller Farm (CMF‐5) | 6.93 | 0.36 | 6.7 | 4.9 | 117 | 100 | 7.8 | 0.51 | 13 | 0.253 | 1 | 1 |
| 58 | 1989 M=6.9 Loma Prieta ‐ Oct 18 | Miller Farm (CMF‐8) | 6.93 | 0.36 | 6 | 4.9 | 110 | 99 | 4.73 | 0.51 | 25 | 0.242 | 1 | 1 |
| 59 | 1989 M=6.9 Loma Prieta ‐ Oct 18 | Miller Farm (CMF‐10) | 6.93 | 0.36 | 8.2 | 3 | 153 | 102 | 6.8 | 1.03 | 20 | 0.313 | 0 | 0 |
| 60 | 1989 M=6.9 Loma Prieta ‐ Oct 18 | Farris Farm (FAR‐58) | 6.93 | 0.36 | 7.4 | 4.8 | 136 | 111 | 11.07 | 0.51 | 4 | 0.261 | 1 | 1 |
| 61 | 1989 M=6.9 Loma Prieta ‐ Oct 18 | Farris Farm (FAR‐59) | 6.93 | 0.36 | 8 | 4.8 | 147 | 116 | 9.4 | 0.51 | 7 | 0.266 | 1 | 1 |
| 62 | 1989 M=6.9 Loma Prieta ‐ Oct 18 | Farris Farm (FAR‐61) | 6.93 | 0.36 | 7.4 | 4.2 | 136 | 105 | 7.75 | 0.65 | 11 | 0.275 | 1 | 1 |
| 63 | 1989 M=6.9 Loma Prieta ‐ Oct 18 | Leonardini (LEN‐37) | 6.93 | 0.22 | 4.9 | 2.5 | 91 | 67 | 3.45 | 0.51 | 12 | 0.183 | 0 | 1 |
| 64 | 1989 M=6.9 Loma Prieta ‐ Oct 18 | Leonardini (LEN‐39) | 6.93 | 0.22 | 2.9 | 1.9 | 53 | 43 | 2.47 | 0.24 | 11 | 0.171 | 1 | 1 |

**Table 1** (continued)

| **No** | **Earthquake** | **Site** | **M_v_** | **a_max_ (g)** | **d**  **(m)** | **dw**  **(m)** | **σvc (kPa)** | **σ'vc (kPa)** | **qc (MPa)** | **Rf (%)** | **FC**  **(%)** | **(τav/σ')** | **Field (Yes=1, No=0)** | **ENN (Yes=1, No=0)** |
| --- | --- | --- | --- | --- | --- | --- | --- | --- | --- | --- | --- | --- | --- | --- |
| 65 | 1989 M=6.9 Loma Prieta ‐ Oct 18 | Leonardini (LEN‐51) | 6.93 | 0.22 | 4.7 | 1.8 | 87 | 59 | 4.5 | 0.51 | 10 | 0.201 | 1 | 1 |
| 66 | 1989 M=6.9 Loma Prieta ‐ Oct 18 | Leonardini (LEN‐52a) | 6.93 | 0.22 | 3.2 | 2.7 | 58 | 53 | 6 | 0.61 | 12 | 0.152 | 0 | 0 |
| 67 | 1989 M=6.9 Loma Prieta ‐ Oct 18 | Leonardini (LEN‐53) | 6.93 | 0.22 | 4.3 | 2.1 | 79 | 58 | 5.2 | 0.51 | 9 | 0.187 | 1 | 1 |
| 68 | 1989 M=6.9 Loma Prieta ‐ Oct 18 | Sea Mist (SEA‐31) | 6.93 | 0.22 | 3.5 | 0.8 | 66 | 39 | 2.5 | 0.51 | 24 | 0.232 | 1 | 1 |
| 69 | 1989 M=6.9 Loma Prieta ‐ Oct 18 | Jefferson Ranch (JRR‐141) | 6.93 | 0.21 | 6 | 2.1 | 112 | 73 | 4.2 | 0.51 | 10 | 0.193 | 1 | 1 |
| 70 | 1989 M=6.9 Loma Prieta ‐ Oct 18 | Jefferson Ranch (JRR‐148) | 6.93 | 0.21 | 7.3 | 3 | 135 | 93 | 8.5 | 0.51 | 5 | 0.179 | 1 | 1 |
| 71 | 1989 M=6.9 Loma Prieta ‐ Oct 18 | Pajaro Dunes (PD1‐44) | 6.93 | 0.22 | 4.5 | 3.4 | 82 | 72 | 7.8 | 0.51 | 4 | 0.157 | 1 | 0 |
| 72 | 1989 M=6.9 Loma Prieta ‐ Oct 18 | Radovich (RAD‐98) | 6.93 | 0.38 | 5.3 | 3.5 | 97 | 79 | 7.73 | 0.67 | 9 | 0.283 | 0 | 1 |
| 73 | 1989 M=6.9 Loma Prieta ‐ Oct 18 | Marinovich (MRR‐65) | 6.93 | 0.4 | 8.5 | 5.6 | 156 | 128 | 7.6 | 0.71 | 12 | 0.281 | 1 | 1 |
| 74 | 1989 M=6.9 Loma Prieta ‐ Oct 18 | Marinovich (MRR‐67) | 6.93 | 0.4 | 6.5 | 6.2 | 118 | 115 | 14.4 | 0.81 | 15 | 0.245 | 0 | 0 |
| 75 | 1989 M=6.9 Loma Prieta ‐ Oct 18 | Tanimura (TAN‐103) | 6.93 | 0.13 | 8.7 | 5 | 160 | 124 | 4 | 0.62 | 13 | 0.096 | 1 | 1 |
| 76 | 1989 M=6.9 Loma Prieta ‐ Oct 18 | Tanimura (TAN‐105) | 6.93 | 0.13 | 5.3 | 4.2 | 97 | 86 | 3.8 | 0.51 | 30 | 0.089 | 0 | 0 |
| 77 | 1989 M=6.9 Loma Prieta ‐ Oct 18 | McGowan Farm (MCG‐136) | 6.93 | 0.26 | 4 | 2.4 | 74 | 58 | 4 | 0.81 | 15 | 0.206 | 0 | 1 |
| 78 | 1989 M=6.9 Loma Prieta ‐ Oct 18 | McGowan Farm (MCG‐138) | 6.93 | 0.26 | 2.9 | 1.8 | 53 | 43 | 2.17 | 0.72 | 42 | 0.206 | 0 | 1 |
| 79 | 1989 M=6.9 Loma Prieta ‐ Oct 18 | Granite (GRA‐123) | 6.93 | 0.34 | 7.5 | 5 | 138 | 113 | 4.4 | 0.67 | 18 | 0.243 | 1 | 1 |
| 80 | 1989 M=6.9 Loma Prieta ‐ Oct 18 | Southern Pacific Bridge (SPR‐48) | 6.93 | 0.33 | 7 | 5.3 | 129 | 112 | 5.13 | 0.79 | 13 | 0.225 | 1 | 1 |
| 81 | 1989 M=6.9 Loma Prieta ‐ Oct 18 | Silliman (SIL‐68) | 6.93 | 0.38 | 5.2 | 3.5 | 95 | 79 | 5.6 | 0.66 | 18 | 0.282 | 1 | 1 |
| 82 | 1989 M=6.9 Loma Prieta ‐ Oct 18 | KETT (KET‐74) | 6.93 | 0.47 | 2.7 | 1.5 | 50 | 38 | 4.8 | 1.31 | 15 | 0.391 | 1 | 1 |
| 83 | 1989 M=6.9 Loma Prieta ‐ Oct 18 | Martella (MAR‐110) | 6.93 | 0.13 | 4.5 | 1.8 | 84 | 57 | 3.6 | 0.61 | 14 | 0.118 | 0 | 0 |
| 84 | 1989 M=6.9 Loma Prieta ‐ Oct 18 | Martella (MAR‐111) | 6.93 | 0.13 | 4 | 1.7 | 74 | 52 | 4 | 0.61 | 10 | 0.116 | 0 | 0 |
| 85 | 1989 M=6.9 Loma Prieta ‐ Oct 18 | Salinas River Bridge (SRB‐116) | 6.93 | 0.12 | 6.9 | 6.4 | 125 | 120 | 4 | 2.37 | 7 | 0.074 | 0 | 0 |
| 86 | 1989 M=6.9 Loma Prieta ‐ Oct 18 | Salinas River Bridge (SRB‐117) | 6.93 | 0.12 | 7.2 | 6.4 | 131 | 123 | 5.6 | 1.64 | 9 | 0.075 | 0 | 0 |
| 87 | 1989 M=6.9 Loma Prieta ‐ Oct 18 | Alameda Bay Farm Isl. (HBI‐ P6) | 6.93 | 0.24 | 3.8 | 3 | 66 | 59 | 6 | 0.37 | 4 | 0.169 | 1 | 1 |
| 88 | 1989 M=6.9 Loma Prieta ‐ Oct 18 | Alameda Bay Farm Isl. (Dike) | 6.93 | 0.24 | 6.2 | 5.5 | 108 | 102 | 10.5 | 2.83 | 10 | 0.154 | 0 | 0 |
| 89 | 1989 M=6.9 Loma Prieta ‐ Oct 18 | POO7‐2 | 6.93 | 0.28 | 6.3 | 3 | 116 | 84 | 5.5 | 0.41 | 3 | 0.232 | 1 | 1 |
| 90 | 1989 M=6.9 Loma Prieta ‐ Oct 18 | POO7‐3 | 6.93 | 0.28 | 6.3 | 3 | 116 | 84 | 5.6 | 1.53 | 3 | 0.232 | 1 | 1 |
| 91 | 1989 M=6.9 Loma Prieta ‐ Oct 18 | POR‐2&3&4 | 6.93 | 0.18 | 5.5 | 3.5 | 89 | 70 | 1.9 | 0.58 | 52 | 0.14 | 1 | 1 |
| 92 | 1989 M=6.9 Loma Prieta ‐ Oct 18 | SFOBB‐1 | 6.93 | 0.28 | 6.3 | 3 | 117 | 84 | 5.2 | 0.49 | 8 | 0.233 | 1 | 1 |
| 93 | 1989 M=6.9 Loma Prieta ‐ Oct 18 | SFOBB‐2 | 6.93 | 0.28 | 7.5 | 3 | 139 | 95 | 8.1 | 0.44 | 12 | 0.241 | 1 | 1 |
| 94 | 1989 M=6.9 Loma Prieta ‐ Oct 18 | Treasure Island Fire Station | 6.93 | 0.16 | 5 | 1.5 | 93 | 59 | 4 | 0.72 | 20 | 0.155 | 1 | 0 |
| 95 | 1994 M=6.7 Northridge ‐ Jan 17 | Balboa Blvd Unit C (BAL‐10) | 6.69 | 0.84 | 8.9 | 7.2 | 164 | 147 | 7.6 | 2.5 | 50 | 0.526 | 1 | 1 |

**Table 1** (continued)

| **No** | **Earthquake** | **Site** | **M_v_** | **a_max_ (g)** | **d**  **(m)** | **dw**  **(m)** | **σvc (kPa)** | **σ'vc (kPa)** | **qc (MPa)** | **Rf (%)** | **FC**  **(%)** | **(τav/σ')** | **Field (Yes=1, No=0)** | **ENN (Yes=1, No=0)** |
| --- | --- | --- | --- | --- | --- | --- | --- | --- | --- | --- | --- | --- | --- | --- |
| 96 | 1994 M=6.7 Northridge ‐ Jan 17 | Rory Lane (M‐27) | 6.69 | 0.8 | 3.9 | 3.4 | 71 | 66 | 8 | 1.2 | 20 | 0.534 | 1 | 1 |
| 97 | 1995 M=6.9 Hyogoken‐Nambu ‐ Jan 16 | Kobe Customs Maya Office B | 6.9 | 0.6 | 4.5 | 1.8 | 84 | 57 | 5.02 | 0.89 | 25 | 0.543 | 1 | 1 |
| 98 | 1995 M=6.9 Hyogoken‐Nambu ‐ Jan 16 | Kobe Port Const. Office | 6.9 | 0.6 | 4 | 2.5 | 72 | 57 | 3.79 | 0.3 | 15 | 0.471 | 1 | 1 |
| 99 | 1995 M=6.9 Hyogoken‐Nambu ‐ Jan 16 | Kobe Wharf Public Co. | 6.9 | 0.45 | 4.8 | 2.1 | 86 | 60 | 4.28 | 0.79 | 28 | 0.396 | 1 | 1 |
| 100 | 1995 M=6.9 Hyogoken‐Nambu ‐ Jan 16 | Koyo Pump Station | 6.9 | 0.45 | 6 | 2.6 | 109 | 76 | 2 | 1.8 | 70 | 0.391 | 1 | 1 |
| 101 | 1995 M=6.9 Hyogoken‐Nambu ‐ Jan 16 | Mizukasa Park | 6.9 | 0.65 | 4.3 | 2 | 77 | 55 | 8.8 | 0.46 | 0 | 0.565 | 1 | 1 |
| 102 | 1995 M=6.9 Hyogoken‐Nambu ‐ Jan 16 | Nagashi Park | 6.9 | 0.65 | 1.4 | 1 | 25 | 21 | 7.9 | 0.39 | 0 | 0.498 | 0 | 0 |
| 103 | 1995 M=6.9 Hyogoken‐Nambu ‐ Jan 16 | New Port No. 6 Pier | 6.9 | 0.6 | 4.5 | 2.5 | 81 | 61 | 6.21 | 0.44 | 7 | 0.49 | 1 | 1 |
| 104 | 1995 M=6.9 Hyogoken‐Nambu ‐ Jan 16 | New Wharf Const. Offices | 6.9 | 0.45 | 3.5 | 2.6 | 62 | 53 | 2.3 | 0.96 | 48 | 0.329 | 1 | 1 |
| 105 | 1995 M=6.9 Hyogoken‐Nambu ‐ Jan 16 | Nisseki Kobe Oil Tank A | 6.9 | 0.6 | 5.5 | 2.4 | 99 | 69 | 4.02 | 0.62 | 28 | 0.523 | 1 | 1 |
| 106 | 1995 M=6.9 Hyogoken‐Nambu ‐ Jan 16 | Nisseki Kobe Oil Tank B | 6.9 | 0.6 | 5.5 | 2.4 | 99 | 69 | 4.79 | 0.75 | 26 | 0.523 | 1 | 1 |
| 107 | 1995 M=6.9 Hyogoken‐Nambu ‐ Jan 16 | Shimonakajima Park | 6.9 | 0.65 | 3.8 | 2 | 68 | 51 | 12.68 | 0.75 | 0 | 0.545 | 0 | 0 |
| 108 | 1995 M=6.9 Hyogoken‐Nambu ‐ Jan 16 | Shiporex Kogyo Osaka Factory | 6.9 | 0.4 | 5.5 | 1.5 | 102 | 62 | 2.48 | 0.43 | 36 | 0.396 | 1 | 1 |
| 109 | 1995 M=6.9 Hyogoken‐Nambu ‐ Jan 16 | Sumiyoshi Elementary | 6.9 | 0.6 | 2.8 | 1.9 | 50 | 41 | 11 | 0.61 | 0 | 0.462 | 0 | 0 |
| 110 | 1995 M=6.9 Hyogoken‐Nambu ‐ Jan 16 | Taito Kobe Factory | 6.9 | 0.45 | 3.7 | 1.6 | 67 | 47 | 2.5 | 0.41 | 30 | 0.406 | 1 | 1 |
| 111 | 1995 M=6.9 Hyogoken‐Nambu ‐ Jan 16 | Tokuyama Concrete Factory | 6.9 | 0.5 | 5.2 | 2 | 95 | 64 | 2.96 | 0.44 | 31 | 0.456 | 1 | 1 |
| 112 | 1995 M=6.9 Hyogoken‐Nambu ‐ Jan 16 | Yoshida Kogyo Factory | 6.9 | 0.5 | 4.1 | 3 | 73 | 62 | 12.8 | 2.91 | 31 | 0.365 | 0 | 0 |
| 113 | 1999 M=7.5 Kocaeli ‐ Aug 17 | Adapazari Site B | 7.51 | 0.4 | 3.8 | 3.3 | 68 | 63 | 6.2 | 0.52 | 35 | 0.273 | 1 | 1 |
| 114 | 1999 M=7.5 Kocaeli ‐ Aug 17 | Adapazari Site C2 | 7.51 | 0.4 | 3.7 | 0.4 | 69 | 37 | 2.9 | 0.81 | 35 | 0.47 | 1 | 1 |
| 115 | 1999 M=7.5 Kocaeli ‐ Aug 17 | Adapazari Site D | 7.51 | 0.4 | 2.2 | 1.5 | 39 | 32 | 1.25 | 0.59 | 65 | 0.308 | 1 | 1 |
| 116 | 1999 M=7.5 Kocaeli ‐ Aug 17 | Adapazari Site F | 7.51 | 0.4 | 2.4 | 0.5 | 45 | 26 | 3.32 | 0.3 | 42 | 0.44 | 1 | 1 |
| 117 | 1999 M=7.5 Kocaeli ‐ Aug 17 | Adapazari Site G | 7.51 | 0.4 | 2.1 | 0.5 | 39 | 23 | 2.49 | 0.32 | 65 | 0.439 | 1 | 1 |
| 118 | 1999 M=7.5 Kocaeli ‐ Aug 17 | Adapazari Site H | 7.51 | 0.4 | 2.5 | 1.7 | 45 | 37 | 2.6 | 0.59 | 15 | 0.311 | 1 | 1 |
| 119 | 1999 M=7.5 Kocaeli ‐ Aug 17 | Adapazari Site J | 7.51 | 0.4 | 2.5 | 0.6 | 46 | 28 | 1.5 | 0.87 | 82 | 0.428 | 1 | 1 |
| 120 | 1999 M=7.5 Kocaeli ‐ Aug 17 | Degirmendere DN‐1 | 7.51 | 0.4 | 2.2 | 1.7 | 38 | 34 | 7.11 | 0.99 | 12 | 0.291 | 1 | 1 |
| 121 | 1999 M=7.5 Kocaeli ‐ Aug 17 | Degirmendere DN‐2 | 7.51 | 0.4 | 3.2 | 2.5 | 57 | 50 | 7.8 | 1.13 | 12 | 0.29 | 0 | 0 |
| 122 | 1999 M=7.5 Kocaeli ‐ Aug 17 | Yalova Harbor | 7.51 | 0.3 | 4.9 | 0.8 | 92 | 51 | 5.23 | 0.47 | 11 | 0.338 | 1 | 1 |
| 123 | 1999 M=7.6 Chi‐Chi ‐ Sept 20 | Nantou Site C, NCC‐1,2,&3 | 7.62 | 0.38 | 2.5 | 1 | 46 | 31 | 2 | 1.02 | 38 | 0.36 | 1 | 1 |
| 124 | 1999 M=7.6 Chi‐Chi ‐ Sept 20 | Yuanlin C‐4 | 7.62 | 0.25 | 4.5 | 0.7 | 84 | 46 | 3 | 1.3 | 45 | 0.286 | 1 | 1 |
| 125 | 1999 M=7.6 Chi‐Chi ‐ Sept 20 | Yuanlini C‐19 | 7.62 | 0.25 | 5 | 0.6 | 94 | 50 | 2 | 1.05 | 54 | 0.293 | 1 | 1 |
| 126 | 1999 M=7.6 Chi‐Chi ‐ Sept 20 | Yuanlin C‐22 | 7.62 | 0.25 | 3.5 | 1.1 | 64 | 41 | 2.7 | 0.46 | 27 | 0.249 | 1 | 1 |
| 127 | 1999 M=7.6 Chi‐Chi ‐ Sept 20 | Yuanlin C‐24 | 7.62 | 0.25 | 6.5 | 1.2 | 121 | 69 | 3.8 | 0.62 | 30 | 0.27 | 1 | 1 |
| 128 | 1999 M=7.6 Chi‐Chi ‐ Sept 20 | Yuanlin C‐25 | 7.62 | 0.25 | 5.5 | 3.5 | 98 | 79 | 2 | 1.05 | 61 | 0.195 | 1 | 1 |
| 129 | 1999 M=7.6 Chi‐Chi ‐ Sept 20 | Yuanlin C‐32 | 7.62 | 0.25 | 6 | 0.7 | 112 | 61 | 3.4 | 0.64 | 32 | 0.287 | 1 | 1 |

**Table 1** (continued)

| **No** | **Earthquake** | **Site** | **M_v_** | **a_max_ (g)** | **d**  **(m)** | **dw**  **(m)** | **σvc (kPa)** | **σ'vc (kPa)** | **qc (MPa)** | **Rf (%)** | **FC**  **(%)** | **(τav/σ')** | **Field (Yes=1, No=0)** | **ENN (Yes=1, No=0)** |
| --- | --- | --- | --- | --- | --- | --- | --- | --- | --- | --- | --- | --- | --- | --- |
| 130 | 2010 M=7.1 Darfield ‐ Sept 4 | SHY‐09 | 7 | 0.187 | 4.8 | 2 | 88 | 61 | 3.7 | 0.19 | 4 | 0.167 | 0 | 0 |
| 131 | 2011 M‐6.2 Christchurch ‐ Feb 22 | SHY‐09 | 6.2 | 0.347 | 4.8 | 2 | 88 | 61 | 3.7 | 0.19 | 4 | 0.303 | 1 | 1 |
| 132 | 2010 M=7.1 Darfield ‐ Sept 4 | AVD‐07 | 7 | 0.183 | 3.7 | 1.7 | 66 | 48 | 6.9 | 0.64 | 7 | 0.158 | 0 | 0 |
| 133 | 2011 M‐6.2 Christchurch ‐ Feb 22 | AVD‐07 | 6.2 | 0.396 | 3.7 | 1.7 | 66 | 48 | 6.9 | 0.64 | 7 | 0.336 | 1 | 1 |
| 134 | 2010 M=7.1 Darfield ‐ Sept 4 | BUR‐46 | 7 | 0.167 | 7.3 | 1.3 | 138 | 80 | 7.37 | 0.61 | 4 | 0.171 | 1 | 1 |
| 135 | 2011 M‐6.2 Christchurch ‐ Feb 22 | BUR‐46 | 6.2 | 0.323 | 7.3 | 1.3 | 138 | 80 | 7.37 | 0.61 | 4 | 0.318 | 1 | 1 |
| 136 | 2010 M=7.1 Darfield ‐ Sept 4 | CBD‐21 | 7 | 0.219 | 5.5 | 1.4 | 104 | 64 | 12.09 | 0.58 | 0 | 0.218 | 0 | 0 |
| 137 | 2011 M‐6.2 Christchurch ‐ Feb 22 | CBD‐21 | 6.2 | 0.46 | 5.5 | 1.4 | 104 | 64 | 12.09 | 0.58 | 0 | 0.446 | 1 | 1 |
| 138 | 2010 M=7.1 Darfield ‐ Sept 4 | FND‐01 | 7 | 0.199 | 3.8 | 1.8 | 67 | 48 | 2.8 | 0.69 | 24 | 0.173 | 1 | 1 |
| 139 | 2011 M‐6.2 Christchurch ‐ Feb 22 | FND‐01 | 6.2 | 0.382 | 3.8 | 1.8 | 67 | 48 | 2.8 | 0.69 | 24 | 0.327 | 1 | 1 |
| 140 | 2010 M=7.1 Darfield ‐ Sept 4 | KAN‐03 | 7 | 0.237 | 5.2 | 1 | 100 | 58 | 8.21 | 0.39 | 3 | 0.249 | 1 | 1 |
| 141 | 2011 M‐6.2 Christchurch ‐ Feb 22 | KAN‐03 | 6.2 | 0.188 | 5.2 | 1 | 100 | 58 | 8.21 | 0.39 | 3 | 0.193 | 0 | 0 |
| 142 | 2010 M=7.1 Darfield ‐ Sept 4 | KAN‐05 | 7 | 0.227 | 3.6 | 2 | 66 | 50 | 3.78 | 0.46 | 8 | 0.189 | 1 | 1 |
| 143 | 2011 M‐6.2 Christchurch ‐ Feb 22 | KAN‐05 | 6.2 | 0.183 | 3.6 | 2 | 66 | 50 | 3.78 | 0.46 | 8 | 0.15 | 1 | 1 |
| 144 | 2010 M=7.1 Darfield ‐ Sept 4 | KAN‐09 | 7 | 0.239 | 1.9 | 0.9 | 34 | 25 | 2.89 | 0.33 | 9 | 0.212 | 1 | 1 |
| 145 | 2011 M‐6.2 Christchurch ‐ Feb 22 | KAN‐09 | 6.2 | 0.186 | 1.9 | 0.9 | 34 | 25 | 2.89 | 0.33 | 9 | 0.164 | 0 | 1 |
| 146 | 2010 M=7.1 Darfield ‐ Sept 4 | KAN‐19 | 7 | 0.234 | 3.7 | 0.8 | 70 | 42 | 7.75 | 0.53 | 2 | 0.247 | 1 | 1 |
| 147 | 2010 M=7.1 Darfield ‐ Sept 4 | KAN‐23 | 7 | 0.216 | 4.8 | 0.5 | 92 | 50 | 9.33 | 0.63 | 0 | 0.245 | 1 | 1 |
| 148 | 2011 M‐6.2 Christchurch ‐ Feb 22 | KAN‐23 | 6.2 | 0.186 | 4.8 | 0.5 | 92 | 50 | 9.33 | 0.63 | 0 | 0.207 | 0 | 0 |
| 149 | 2010 M=7.1 Darfield ‐ Sept 4 | KAN‐26d | 7 | 0.231 | 6.5 | 1.5 | 122 | 74 | 7.85 | 0.43 | 3 | 0.23 | 1 | 1 |
| 150 | 2011 M‐6.2 Christchurch ‐ Feb 22 | KAN‐26c | 6.2 | 0.181 | 2 | 1.5 | 34 | 30 | 2.51 | 0.56 | 22 | 0.132 | 1 | 1 |
| 151 | 2010 M=7.1 Darfield ‐ Sept 4 | KAN‐28 | 7 | 0.231 | 2.6 | 1.4 | 47 | 35 | 4.17 | 0.34 | 5 | 0.195 | 1 | 1 |
| 152 | 2011 M‐6.2 Christchurch ‐ Feb 22 | KAN‐28 | 6.2 | 0.182 | 2.6 | 1.4 | 47 | 35 | 4.17 | 0.34 | 5 | 0.152 | 1 | 1 |
| 153 | 2010 M=7.1 Darfield ‐ Sept 4 | KAS‐08 | 7 | 0.204 | 2 | 1.3 | 35 | 29 | 2.39 | 0.56 | 20 | 0.161 | 1 | 1 |
| 154 | 2011 M‐6.2 Christchurch ‐ Feb 22 | KAS‐08 | 6.2 | 0.186 | 2 | 1.3 | 35 | 29 | 2.39 | 0.56 | 20 | 0.146 | 1 | 1 |
| 155 | 2010 M=7.1 Darfield ‐ Sept 4 | Z2‐6 | 7 | 0.214 | 2.4 | 2 | 42 | 38 | 4.7 | 0.9 | 16 | 0.152 | 0 | 0 |
| 156 | 2011 M‐6.2 Christchurch ‐ Feb 22 | Z2‐6 | 6.2 | 0.451 | 2.4 | 2 | 42 | 38 | 4.7 | 0.9 | 16 | 0.317 | 1 | 1 |
| 157 | 2010 M=7.1 Darfield ‐ Sept 4 | Z4‐4 | 7 | 0.215 | 2.6 | 2 | 46 | 40 | 4.12 | 0.91 | 18 | 0.158 | 0 | 0 |
| 158 | 2011 M‐6.2 Christchurch ‐ Feb 22 | Z4‐4 | 6.2 | 0.45 | 2.6 | 2 | 46 | 40 | 4.12 | 0.91 | 18 | 0.327 | 1 | 1 |
| 159 | 2010 M=7.1 Darfield ‐ Sept 4 | Z8‐11 | 7 | 0.219 | 1.7 | 1.4 | 30 | 27 | 2.3 | 0.75 | 39 | 0.157 | 1 | 1 |
| 160 | 2011 M‐6.2 Christchurch ‐ Feb 22 | Z8‐11 | 6.2 | 0.453 | 1.7 | 1.4 | 30 | 27 | 2.3 | 0.75 | 39 | 0.322 | 1 | 1 |
| 161 | 2011 M=9.0 Tohoku ‐ Mar 11 | Hinode Minami ES | 9 | 0.17 | 4.8 | 1.1 | 89 | 53 | 10.1 | 0.8 | 3 | 0.187 | 0 | 0 |
| 162 | 2011 M=9.0 Tohoku ‐ Mar 11 | Hosoyama Nekki | 9 | 0.18 | 4.2 | 2.5 | 76 | 60 | 6.6 | 0.4 | 5 | 0.15 | 0 | 0 |
| 163 | 2011 M=9.0 Tohoku ‐ Mar 11 | Takasu Chuou Park | 9 | 0.21 | 6.8 | 1.1 | 127 | 71 | 6.7 | 0.7 | 15 | 0.245 | 1 | 1 |
| 164 | 2011 M=9.0 Tohoku ‐ Mar 11 | Takasu Kaihin Park | 9 | 0.22 | 9 | 1.3 | 169 | 93 | 9.5 | 0.5 | 2 | 0.261 | 1 | 1 |
| 165 | 2011 M=9.0 Tohoku ‐ Mar 11 | Akemi ES | 9 | 0.169 | 10.1 | 1.2 | 189 | 102 | 8.3 | 0.6 | 11 | 0.205 | 0 | 0 |
| 166 | 1989 M=6.9 Loma Prieta ‐ Oct 18 | MBARI No. 3 (RC‐6) | 6.93 | 0.28 | 4.1 | 2.6 | 73 | 59 | 13 | 0.2 | 1 | 0.218 | 0 | 0 |
| 167 | 1989 M=6.9 Loma Prieta ‐ Oct 18 | MBARI No. 3 (RC‐7) | 6.93 | 0.28 | 4.7 | 3.7 | 83 | 73 | 9.2 | 0.25 | 1 | 0.196 | 0 | 0 |

**Table 1** (continued)

| **No** | **Earthquake** | **Site** | **M_v_** | **a_max_ (g)** | **d**  **(m)** | **dw**  **(m)** | **σvc (kPa)** | **σ'vc (kPa)** | **qc (MPa)** | **Rf (%)** | **FC**  **(%)** | **(τav/σ')** | **Field (Yes=1, No=0)** | **ENN (Yes=1, No=0)** |
| --- | --- | --- | --- | --- | --- | --- | --- | --- | --- | --- | --- | --- | --- | --- |
| 168 | 1989 M=6.9 Loma Prieta ‐ Oct 18 | MBARI Technology (RC‐9) | 6.93 | 0.28 | 3.5 | 2 | 63 | 48 | 12.4 | 0.25 | 4 | 0.23 | 0 | 0 |
| 169 | 1989 M=6.9 Loma Prieta ‐ Oct 18 | MBARI No. 4 (CPT‐1, 2, 3, & | 6.93 | 0.28 | 3.1 | 1.9 | 56 | 44 | 8.77 | 0.11 | 4 | 0.225 | 0 | 0 |
| 170 | 1989 M=6.9 Loma Prieta ‐ Oct 18 | General Fish (CPT‐5) | 6.93 | 0.28 | 2.1 | 1.5 | 37 | 31 | 2.5 | 0.2 | 4 | 0.213 | 1 | 1 |
| 171 | 1989 M=6.9 Loma Prieta ‐ Oct 18 | General Fish (CPT‐6) | 6.93 | 0.28 | 2.6 | 1.7 | 46 | 38 | 10 | 0.2 | 4 | 0.22 | 0 | 0 |
| 172 | 1989 M=6.9 Loma Prieta ‐ Oct 18 | Harbor Office (UC‐12) | 6.93 | 0.28 | 4.1 | 1.9 | 74 | 53 | 6.2 | 0.91 | 15 | 0.246 | 1 | 1 |
| 173 | 1989 M=6.9 Loma Prieta ‐ Oct 18 | Harbor Office (UC‐13) | 6.93 | 0.28 | 4.1 | 1.9 | 74 | 53 | 4.3 | 1.02 | 15 | 0.246 | 1 | 1 |
| 174 | 1989 M=6.9 Loma Prieta ‐ Oct 18 | Harbor Office (UC‐20) | 6.93 | 0.28 | 4.7 | 3 | 84 | 67 | 4.1 | 0.59 | 10 | 0.215 | 1 | 1 |
| 175 | 1989 M=6.9 Loma Prieta ‐ Oct 18 | Harbor Office (UC‐21) | 6.93 | 0.28 | 4.2 | 2.7 | 75 | 60 | 4.9 | 0.49 | 10 | 0.217 | 1 | 1 |
| 176 | 1989 M=6.9 Loma Prieta ‐ Oct 18 | Woodward Marine (UC‐9) | 6.93 | 0.28 | 2.9 | 1.2 | 53 | 36 | 6.6 | 0.3 | 5 | 0.259 | 1 | 1 |
| 177 | 1989 M=6.9 Loma Prieta ‐ Oct 18 | Woodward Marine (UC‐10) | 6.93 | 0.28 | 2 | 1 | 36 | 26 | 3.1 | 0.4 | 5 | 0.246 | 1 | 1 |
| 178 | 1989 M=6.9 Loma Prieta ‐ Oct 18 | Woodward Marine (UC‐11) | 6.93 | 0.28 | 2.2 | 1 | 40 | 28 | 3.1 | 0.36 | 15 | 0.254 | 1 | 1 |
| 179 | 1995 M=6.9 Hyogoken‐Nambu ‐ Jan 16 | Dust Management Center | 6.9 | 0.37 | 7 | 2 | 125 | 76 | 7.5 | 0.38 | 2 | 0.36 | 1 | 1 |
| 180 | 1995 M=6.9 Hyogoken‐Nambu ‐ Jan 16 | Fukuzumi Park | 6.9 | 0.65 | 11.8 | 3.1 | 210 | 125 | 17.87 | 1.44 | 13 | 0.583 | 0 | 0 |
| 181 | 1995 M=6.9 Hyogoken‐Nambu ‐ Jan 16 | Hamakoshienn Housing Area | 6.9 | 0.5 | 3.8 | 2 | 67 | 50 | 4.59 | 0.66 | 20 | 0.421 | 1 | 1 |
| 182 | 1995 M=6.9 Hyogoken‐Nambu ‐ Jan 16 | Honjyo Central Park | 6.9 | 0.7 | 5 | 2.5 | 93 | 68 | 14.4 | 0.6 | 0 | 0.584 | 0 | 0 |
| 183 | 1995 M=6.9 Hyogoken‐Nambu ‐ Jan 16 | Imazu Elementary School | 6.9 | 0.6 | 4.6 | 1.4 | 86 | 54 | 10 | 0.15 | 0 | 0.584 | 1 | 1 |
| 184 | 1995 M=6.9 Hyogoken‐Nambu ‐ Jan 16 | Kobe Art Institute | 6.9 | 0.5 | 3.4 | 3 | 62 | 58 | 12.83 | 1.55 | 13 | 0.335 | 0 | 0 |
| 185 | 1995 M=6.9 Hyogoken‐Nambu ‐ Jan 16 | Kobe Customs Maya Office A | 6.9 | 0.6 | 6.5 | 1.8 | 121 | 75 | 2.32 | 0.42 | 41 | 0.578 | 1 | 1 |
| 186 | 1999 M=7.5 Kocaeli ‐ Aug 17 | Hotel Sapanca SH‐4 | 7.51 | 0.37 | 1.6 | 0.5 | 30 | 19 | 0.94 | 0.46 | 5 | 0.376 | 1 | 1 |
| 187 | 1976 M=7.6 Tangshan ‐ July 27 | T11 ‐ Tangshan | 7.6 | 0.61 | 2 | 0.9 | 36 | 24 | 3.8 | 2.12 | 9 | 0.576 | 1 | 1 |
| 188 |  | T13 ‐ Tangshan | 7.6 | 0.58 | 5.6 | 1.1 | 104 | 60 | 11.3 | 0.97 | 5 | 0.632 | 1 | 1 |
| 189 |  | T16 ‐ Tangshan | 7.6 | 0.26 | 6.6 | 3.5 | 119 | 89 | 15.5 | 0.98 | 2 | 0.215 | 0 | 0 |
| 190 |  | T19 ‐ Tangshan | 7.6 | 0.25 | 3.3 | 1.1 | 60 | 39 | 4.1 | 0.4 | 5 | 0.247 | 1 | 1 |
| 191 |  | T21 ‐ Tangshan | 7.6 | 0.25 | 3.6 | 3.1 | 62 | 58 | 10 | 0.38 | 5 | 0.171 | 0 | 0 |
| 192 | 1999 M=7.6 Chi‐Chi ‐ Sept 20 | Nantou Site C‐8 | 7.62 | 0.38 | 7 | 1 | 131 | 72 | 2.72 | 2.16 | 65 | 0.423 | 1 | 1 |
| 193 | 1999 M=7.6 Chi‐Chi ‐ Sept 20 | WuFeng Site B | 7.62 | 0.6 | 3 | 1.1 | 55 | 36 | 3 | 1.02 | 35 | 0.578 | 1 | 1 |
| 194 | 1999 M=7.6 Chi‐Chi ‐ Sept 20 | WuFeng Site C | 7.62 | 0.6 | 4.5 | 1.2 | 83 | 51 | 3.5 | 2.05 | 14 | 0.619 | 1 | 1 |
| 195 | 2010 M=7.1 Darfield ‐ Sept 4 | KAS‐11 | 7 | 0.211 | 2.6 | 1.2 | 47 | 34 | 3.9 | 0.6 | 10 | 0.187 | 1 | 1 |
| 196 | 2011 M‐6.2 Christchurch ‐ Feb 22 | KAS‐11 | 6.2 | 0.186 | 2.6 | 1.2 | 47 | 34 | 3.9 | 0.6 | 10 | 0.164 | 1 | 1 |
| 197 | 1987 M=6.6 Edgecumbe, NZ ‐ Mar 2 | Brady Farm BDY004 | 6.6 | 0.4 | 4.2 | 1.5 | 77 | 51 | 7.53 | 0.41 | 15 | 0.373 | 0 | 0 |
| 198 | 1987 M=6.5 Superstition Hills 02 ‐ Nov 24 | Wildlife B | 6.54 | 0.206 | 4.8 | 1.2 | 90 | 54 | 5.33 | 1.5 | 30 | 0.208 | 1 | 1 |
| 199 | 2010 M=7.1 Darfield ‐ Sept 4 | KAS‐40 | 7 | 0.224 | 2.3 | 1.9 | 41 | 36 | 3.52 | 0.35 | 8 | 0.16 | 1 | 1 |
| 200 | 2010 M=7.1 Darfield ‐ Sept 4 | RCH‐14 | 7 | 0.183 | 4.5 | 2.3 | 81 | 60 | 2.38 | 0.03 | 7 | 0.154 | 0 | 0 |
